# Examining procedural fairness in anti-doping disputes: a comparative empirical analysis

**DOI:** 10.1007/s40318-022-00222-5

**Published:** 2022-06-10

**Authors:** Shaun Star, Sarah Kelly

**Affiliations:** 1 Professor, Jindal Global Law School, O.P. Jindal Global University, Sonipat, Haryana India; 2 PhD Candidate, TC Beirne School of Law, The University of Queensland, St Lucia, QLD Australia; 3 Associate Professor, University of Queensland Business School, University of Queensland, St Lucia, QLD Australia

**Keywords:** World Anti-Doping Code, National Anti-Doping Organizations (NADOs), Due process, Empirical legal research, Access to justice, Delay

## Abstract

While the principles of procedural fairness apply in anti-doping disputes pursuant to Article 8 of the Word Anti-Doping Code, 2021 (the Code), there has been limited research assessing whether due process requirements are applied consistently by national anti-doping tribunals. This paper investigates the extent to which the procedural requirements set out under the Code are followed in practice, with a focus on India, New Zealand and Canada, facilitating comparison between developed and developing jurisdictions. By providing an evidence-based examination of first instance anti-doping procedures, this study confirms existing theories on the overall lack of harmonization in anti-doping procedures. We undertook a frequency analysis on the full-text awards handed down by first instance anti-doping tribunals in the comparative jurisdictions and the findings highlight inconsistent application of timeliness requirements and access to legal representation. Critically, in India, disputes take significantly longer to be resolved than in Canada and New Zealand, while far fewer Indian athletes are represented by legal counsel. In all jurisdictions, athletes who were represented by counsel were more likely to see a reduction in their sanctions. The study provides empirical evidence of systemic issues associated with timeliness and access to justice in anti-doping tribunals across jurisdictions and reinforces the need to focus on capacity building and enforcement of procedural safeguards, especially in developing countries. Practical recommendations include strategies to better achieve compliance and harmonization in protecting the procedural rights of athletes, particularly those athletes affected by the current application of the Code where cultural and socio-economic barriers may exacerbate procedural issues.

## Introduction

Athletes are provided minimum procedural guarantees under the World Anti-Doping Code (the Code) and the International Standard of Results Management (ISRM) when they are before a dispute resolution tribunal for an alleged anti-doping violation. The principle of procedural fairness (or due process) has been applied by the Court of Arbitration for Sport (CAS) and first instance tribunals across jurisdictions.^1^ However, it has been argued that the extent to which different jurisdictions comply with procedural safeguards varies, especially when comparing developed and developing countries.^2^ Despite the quest for harmonization in the application and compliance of the Code,^3^ there has been a dearth of empirical research to assess whether the Code has been applied consistently by National Anti-Doping Organizations (NADOs) and national tribunals. This paper investigates the extent to which the procedural requirements set out under the Code are followed in practice. Given that the vast majority of athletes have their anti-doping violations heard by national tribunals at first instance, this paper will explore whether the elements of procedural fairness have been applied in practice before these national tribunals, with a particular focus on India, New Zealand and Canada, allowing for a comparison between developed and developing jurisdictions.

Some commentators argue that while the one-size-fits-all approach of the Code aims to encourage harmonization and fair play in the application of anti-doping rules, it also inadvertently promotes inequality between developed and developing nations.^4^ To test this theory, we conduct a frequency analysis of cases to assess national compliance with the elements of procedural fairness enshrined in Article 8 of the Code, including timeliness and access to legal representation. While studies have been conducted to assess compliance with procedural fairness requirements, such as timeliness of disputes and access to legal representation in courts and tribunals,^5^ such studies are yet to be conducted in an anti-doping context despite calls to reinforce evidence-based policies in anti-doping procedures.^6^

Adopting procedural fairness (due process) as a theoretical framework, the authors aim to empirically assess the theories (and anecdotal evidence) that there are procedural irregularities at first instance doping tribunals,^7^ and that there is a systemic lack of harmonization in anti-doping procedures,^8^ by considering the following research questions:

- How long does it take to resolve anti-doping disputes at first instance national tribunals? Do NADOs and first instance tribunals comply with procedural fairness norms as required under the Code and domestic rules, including adherence to time limits?
- Do access to justice issues exist at a domestic level, and how many athletes are represented by lawyers at first instance hearings as a proxy for access?
- Does access to legal representation impact the outcome of anti-doping proceedings?
- Does compliance with procedural fairness norms vary across developed and developing countries?

Given the allegations of procedural shortcomings in the application of the Code in India,^9^ coupled with the relatively high prevalence of anti-doping within India,^10^ further analysis is warranted into the extent of alleged non-compliance with the procedural safeguards under the Code within its national framework. Commentators have argued that a timely hearing and competent legal representation are important given that an athlete’s career may be in jeopardy if these fundamental procedural guarantees are not afforded to them.^11^ Given previous suggestions that there are inconsistencies in the application of procedural requirements between developing and developed nations,^12^ a comparative analysis with New Zealand and Canadian disputes is conducted. It is argued that the stark contrast between the jurisdictions highlights that there are significant challenges with the World Anti-Doping Agency’s (WADA) quest for harmonization in terms of the implementation of procedural norms under the Code and the NADO rules. Most prior research has examined CAS decisions,^13^ whereas this research focuses on national tribunal decisions at first instance, hypothesizing that these are the foundations of access to justice and consistency in decision-making procedures. This study, therefore, advances the existing knowledge with respect to procedural fairness for athletes in anti-doping at first instance,^14^ which further strengthens the arguments raised with respect to the perceived legitimacy challenges faced by the anti-doping system at large.^15^ Significantly, by applying methodologies used in research on civil and criminal court procedures, this study provides, for the first time, an evidence-based examination of first instance anti-doping procedures in practice, thereby confirming existing theories on the overall lack of harmonization in anti-doping procedures.

To investigate the effectiveness of compliance with the prescribed procedural requirements under the national anti-doping rules, we have conducted a frequency analysis on the full-text awards handed down by first instance anti-doping tribunals in India, Canada and New Zealand from the inception of the Code in 2009 to 2015. The findings make clear that while the Code purports to create a harmonized system of anti-doping disputes, there is a stark contrast between how the tenets of procedural fairness (including timeliness and legal representation) are fulfilled across jurisdictions. In India, for instance, disputes take significantly longer to be resolved than in Canada and New Zealand. In addition, far fewer Indian athletes are represented by legal counsel during their anti-doping proceedings. In all jurisdictions, athletes who were represented by counsel were more likely to see a reduction in their sanctions. This is consistent with the theory espoused by Galanter (1974) which suggests that stronger institutional parties to a dispute have a higher likelihood of success.^16^ Accordingly, we argue that the relatively low levels of legal representation of Indian athletes are a cause of concern.

Many scholars argue that there is scope for further reform to protect the procedural rights of athletes in anti-doping disputes.^17^ However, often reform with respect to dispute resolution procedures has been guided by “impressions and anecdotes”, rather than based on a data-driven approach.^18^ There have, therefore, been recent calls for a stronger evidence-based approach for reforms in anti-doping.^19^ Consequently, this paper has significant practical implications insofar as it provides empirical evidence of systemic issues associated with the implementation of the Code across jurisdictions, and reinforces the need for focus on capacity building and enforcement of procedural safeguards, especially with respect to NADOs and tribunals in developing countries. The recommendations and future research agenda set out throughout this paper suggest strategies to better achieve compliance and harmonization in protecting the procedural rights of athletes, particularly those athletes affected by the current application of the Code in some jurisdictions, especially where cultural and socio-economic barriers may exacerbate procedural issues.

## Anti-doping and the importance of empirical evidence

### Background

The Code is an international set of regulations that regulates anti-doping in sport. The vast majority of countries and international sport governing bodies are governed by the Code.^20^ These countries have ratified the Code into national laws and regulations in their respective jurisdictions, and NADOs have been created to implement the Code domestically. International sporting governing bodies, which are also signatories to the Code, contractually bind athletes and national governments to the Code, to the extent that if they fail to comply with the Code they are unable to participate in sport competitively.^21^ While the Code promotes a harmonized set of rules and regulations for all athletes and national governments, there is a degree of autonomy afforded to national governments with respect to the implementation of certain aspects of the Code.

In India, the NADA Rules (2010 NADA Rules) have been in force since 2010, and these rules were amended in 2015 (2015 NADA Rules) and 2021 (2021 NADA Rules). Under the NADA Rules, anti-doping violations are determined by the Anti-Doping Disciplinary Panel (ADDP) at first instance and the Anti-Doping Appeal Panel (ADAP) on appeal. Since its inception in 2010, ADDP has heard 1206 cases^22^ and ADAP has heard 170 cases.^23^ To date, 14 Indian athletes have had their cases heard by the CAS, only one of which was appealed by the athlete. Therefore, the majority of cases are disposed of by the domestic tribunals in India.

In Canada, the Canadian Anti-Doping Program (CADP), which complies with the Code, is implemented and administered by the Canadian Centre for Ethics in Sport (CCES). The CADP was established in 2009 and has since been revised in 2015 and 2021, to ensure consistency with the Code. Since 2004, anti-doping disputes have been heard by the Sport Dispute Resolution Centre of Canada (SDRCC).

Drug Free Sport New Zealand (DFSNZ) was established under the New Zealand Sports and Drug Agency Act 1994 (subsequently replaced by the Sports Anti-Doping Act 2006) to implement and apply the Code in New Zealand. DFSNZ have amended New Zealand’s Sports Anti-Doping Rules every year over the past decade.^24^ The Sports Tribunal of New Zealand was established in 2003 and it has the authority to hear anti-doping disputes.^25^

The global anti-doping framework requires harmonization and consistency for its legitimacy.^26^ Despite all three jurisdictions adopting the Code, each country has implemented the procedural elements of the Code differently, including with respect to their dispute resolution procedures. Consequently, a comparative study of how anti-doping regulations have been implemented across the three jurisdictions is the focus this study.

### Empirical evidence in anti-doping

The importance of empirical research in legal studies is widely recognized^27^ and has been used increasingly in recent decades.^28^ Empirical legal research can aid our understanding of how the law works in practice,^29^ how courts and tribunals interpret and apply the law, and how legal services are performed.^30^ Such research enables scholars to better understand how the law works in practice, empirically test hypotheses about the law, and identify possible procedural or substantive changes that could improve the law.^31^

Empirical research has been conducted on the prevalence of doping in sport,^32^ and on the impact and perceptions of doping in sport in jurisdictions around the world.^33^ However, despite the importance of empirical research and calls for greater scientific integrity in anti-doping procedures,^34^ there is no published empirical research that focuses on procedural fairness in anti-doping disputes.^35^

In the context of sport arbitration, Lindholm (2019) conducted an empirical analysis on CAS decisions. Lindholm analyses 830 CAS awards and opinions between CAS’s inception and 2014. His publication draws some trends in CAS procedure, including the fact that both CAS arbitrators and litigants are largely from a select group of countries which are not necessarily representative of WADA’s member states. In addition, the study concludes that CAS jurisprudence is consistently applied on a number of critical issues, including *inter alia* the principle of procedural fairness. While Lindholm’s work focuses on CAS decisions more broadly, this paper will focus only on doping disputes conducted in India, New Zealand and Canada by first instance domestic tribunals. The focus on empirically analyzing first instance decisions is particularly important given that the vast majority of athletes do not have the opportunity to have their case heard before the CAS – that is, their cases are ultimately determined by national first instance tribunals.^36^ For instance, while a total of 1206 Indian anti-doping violations have been heard by the national first instance tribunal,^37^ only  14 of these athletes have been appealed to the CAS.^38^

Despite the lack of empirical research in anti-doping proceedings, there has been an increasing amount of empirical research on civil justice systems and administrative tribunals around the world. Such research has helped inform institutional shortcomings in various civil justice systems, and used as evidence of the need for reform in civil and administrative proceedings. For instance, Heise (2000) argues that a “deeper and more systematic understanding of the underlying civil justice system would assist efforts seeking to decrease case disposition time”.^39^ In addition to the issue of timeliness and delay in civil justice proceedings, much research has been conducted on access to justice and legal representation.^40^ Similar research on arbitration proceedings has been conducted in the context of commercial arbitration^41^ and employment arbitration,^42^ but not on anti-doping procedures. Analyzing the procedures within the anti-doping process empirically would be valuable in shaping our understanding of how the implementation of anti-doping rules are applied in practice. This data will also provide much needed evidence to inform discussion about reform in an area that is often debated on the basis of anecdotal evidence without the benefit of such data. This section will investigate how extant empirical legal research in civil litigation has shaped our understanding of procedural fairness of litigants, which can then be applied to the current research context.

### Empirical research in courts and tribunals

#### Delay in courts and tribunals

The present study draws upon existing frameworks from civil and administrative procedure.^43^ Measurements of delay have varied across studies. For instance, several studies have evaluated the duration of litigation from inception to judgment,^44^ while others have examined particular stages of trials in civil litigation.^45^ To date, most scholars who employ empirical research into delay in civil ligation use quantitative research methods that examine existing data sets.^46^ To minimize the resources needed for research, it is common for scholars to draw on publicly available data to analyze delay in civil litigation.^47^

In the existing literature with respect to delay in civil ligation, scholars have undertaken comparative analyses to compare data sets across time.^48^ A number of empirical studies have used descriptive statistics in their analysis of data available from court records. For instance, recent projects have analyzed court records to determine the average number of days of hearings, and the median number of days from filing to the resolution of disputes.^49^

Some scholars have argued that the concept of delay inherently includes subjective and culturally-specific elements, making it difficult to measure empirically,^50^ and as a consequence they may instead use terminology such as “timeliness” to measure whether cases have been resolved within a reasonable time. It has been argued, therefore, that focusing on the “more measurable notion of ‘duration’ of civil proceedings” will enable researchers to more objectively understand the facts that influence case progression.^51^ Scholars have found analyzing the duration of trials particularly valuable from a comparative perspective, as it has enabled them to compare timeliness across jurisdictions,^52^ or even longitudinally within the same jurisdiction.^53^ While such quantitative research is typically analyzed from court records and judgments, further qualitative research may be used to understand the behavioral elements that contribute to delay and supplement the ‘hard’ data on the duration and timeliness of dispute resolution procedures.^54^ Both quantitative and qualitative research, therefore, play an important role in shaping a comprehensive understanding of delay in court proceedings.

#### Access to legal counsel in courts and tribunals

Previous studies have explored parties’ access to legal representation in different domestic contexts. This research has shown that “[a]ccess to lawyers—particularly for people who are currently representing themselves—could significantly change the face of the justice meted out in … civil courts”.^55^ While much of this research has been conducted in the context of civil proceedings, similar research has been conducted in other contexts with similar results. For instance, a study by Lederman and Hrung (2006) conducted an empirical analysis of tax disputes in the USA to understand the “effect the presence of counsel for the taxpayer has both on the financial outcome of the case … and the length of time to settlement or trial”.^56^ The study concluded that:

*…attorneys obtain significantly better results in tried cases than unrepresented taxpayers do-and that the magnitude of that effect increases with greater attorney experience-but, surprisingly, that attorneys do not obtain better outcomes in settled cases. The results also suggest that taxpayers' attorneys do not affect the amount of time Tax Court cases take to settle or go to trial*.^57^

Sandefur (2010) conducted a meta-analysis of existing quantitative research addressing the question “how much does lawyer representation affect who wins and loses in adjudication?”^58^ and concluded that on average, litigants who are represented by lawyers “are more likely to win than are unrepresented people in every study”.^59^ Of the studies analyzed, the extent to which lawyers impacted the likelihood of success varied, from a study where represented litigants were 19% more likely to win, to a study which finds that litigants represented by lawyers were found to be approximately 14 times more likely to win than unrepresented people.^60^ However, the common thread in all studies analyzed was that legal representation greatly increases the likelihood of success of litigants. While research on employment arbitration has also shown that legal representation on average increases the likelihood of success,^61^ similar studies have not yet been conducted in the context of anti-doping arbitration.

There is limited comparative research across jurisdictions.^62^ However, such comparisons can add value in understanding which policies and procedures might influence the procedural nuances in each country, and the relative extent of inconsistency in procedural fairness norms. Comparative approaches also reveal vulnerabilities in the status quo in terms of indirect consequences or inequality elicited by universally applicable codes or policy.

In light of the existing empirical research that has been conducted on civil litigation systems, especially with respect to delay and legal representation, this study will adopt a frequency analysis approach to test the hypothesis that there are disharmony issues concerning procedural fairness in anti-doping cases in first instance proceedings and that these issues are more prevalent in emerging jurisdictions such as India compared to developed nations in the West.

In light of the research questions set out in the introduction, and based on the literature discussed above, the researchers hypothesized that representation by a lawyer will increase the probability of an athlete receiving a more favorable outcome in their anti-doping proceedings. In addition, the researchers predicted that there is a difference between the implementation of the Code in developed and developing countries. In particular, they hypothesized that cases (a) take longer, and (b) fewer athletes are represented by lawyers, in India, as compared to Canada and New Zealand. To test these hypotheses, the study adopted the methodology set out below, replicating the approaches previously applied to civil law contexts.

## Research methodologies

### Data collection and sample

The researchers have sampled awards handed down by anti-doping tribunals in India, Canada and New Zealand. In India, panel and appellate awards handed down between the period 27 May 2009 and 18 December 2015 (the Data Collection Period) from domestic anti-doping tribunals were made available by the Ministry of Youth Affairs and Sport (MYAS). The full-text awards have been studied and analyzed through a frequency analysis. A total of 594 anti-doping rule violation cases (ADRVs) have been collected and collated as part of this sample. According to India’s National Anti-Doping Agency (NADA), 631 anti-doping violations have been determined by the ADDP during the Data Collection Period.^63^ Accordingly, the awards collected and analyzed across the Data Collection Period represent approximately 95 percent of all Indian doping decisions issued during the period in question. A total of 56 ADAP awards from India were also collected and analyzed (i.e., 56 of these cases were appealed during this period) and 10 of these cases were further appealed to the CAS. The researchers have relied on the MYAS for access to the awards handed down by ADDP and ADAP as awards are not publicly available. The fact that such full-text awards are not publicly available is not uncommon in domestic anti-doping proceedings. However, it has been argued that this lack of transparency does in itself present procedural fairness issues as lawyers and athletes do not have access to previous decisions of these anti-doping tribunals.^64^ In addition, the lack of available data and the absence of publicly available full-text awards from first instance doping disputes is a likely reason for the lack of empirical research and public scrutiny of such disputes to date.

The researchers have adopted a convenience sample largely because the available data only pertains to cases up until 2015 (rather than more recent cases) as the awards from more recent Indian cases have not been made available by MYAS. However, the data with respect to compliance with procedural fairness norms is still valuable. The key tenets of procedural fairness, which include timeliness and access to legal representation, have remained largely unchanged within the anti-doping regulations since then. As such, while a longitudinal analysis of how procedural compliance has changed over time may be justified, this study focuses on data available since the early implementation of anti-doping procedures. While procedural requirements were amended in 2015, they did not change fundamentally. However, it should be noted that while the central tenets of procedural fairness have remained the same under the 2021 Code and ISRM,^65^ minimum procedural guarantees have been strengthened, with express time limits expressly enshrined under the ISRM.^66^ In any event, since no similar empirical studies on anti-doping procedure have been conducted to date, it is argued that assessing compliance with procedural fairness norms at first instance hearings will provide a valuable insight into any systemic challenges that may exist in the anti-doping system. If empirical evidence does point to systemic delays in anti-doping proceedings, for example, this research will provide an evidence basis for scrutinizing the need for procedural reform in the implementation of the Code.

To enable a comparative analysis across developed and developing jurisdictions and to determine whether any procedural irregularities are unique to India, cases have also been coded from Canada and New Zealand during the same period. A total of 44 cases from New Zealand and 37 cases from Canada were handed down by first instance domestic tribunals during the Data Collection Period. The samples from New Zealand and Canada are clearly much smaller than that of India, representing that there is a significantly lower number of ADRVs in these countries, a factor of *inter alia* their lower populations.

### Selection of variables

Categories coded as part of the analysis (see below) were preselected with reference to fairness requirements under Article 8 of the Code and with reference to an established framework of procedural fairness, specifically Pound and Clarke’s (2011) commentary of the Code which includes the tenets of:

*timeliness; a fair and impartial hearing panel; the right to be represented by counsel (at the person’s own expense); the right to be informed in a fair and timely manner of the asserted violation; the right to respond to the asserted violation and resulting consequences; the right to present evidence …; the right to an interpreter at the hearing …; and receipt of a timely, written, reasoned decision…*^67^

This commentary largely reflects the procedural safeguards that are still enshrined under more recent versions of the Code, and are therefore accepted as the minimum procedural safeguards to be afforded to athletes in anti-doping procedures.^68^ For the purposes of this study, the researchers have focused on the elements of (i) timeliness; and (ii) access to legal representatives. These are important procedural guarantees under the Code and can be analyzed on the data available in the awards handed down by first instance tribunals. The importance of timeliness and access to counsel have been recognized under the revised 2021 Code and ISRM, and both of these procedural safeguards have been strengthened as a result.^69^ While the other variables are also important, it is typically not possible to collect data with respect to a number of these variables from a review of the awards. In addition, following this preliminary pilot coding approach, consultations were conducted with experienced legal counsel in anti-doping disputes from different jurisdictions to calibrate the final coding categories to apply to the case sample, and these variables were identified by lawyers as particularly important from the athlete’s perspective, thus providing face validity to the proposed coding approach.

The coded variables used in the study, which are set out in Table 1 (Case Variables Coded), were collected for each of the doping violation cases heard by first instance national tribunals in India, Canada and New Zealand during the Data Collection Period.

**Table 1:** Case variables coded

Case Particulars	Case Citation
	Case Name
	Year
	Date – Panel
	Sport
	Gender
	Legal Representation
	Advocate/Lawyer
	Type of ADV
Banned Substance(s)	Anabolic Steroid
	Diuretics
	Stimulant
	Other (e.g., Anabolic Agent or SERM)
Outcome	Decision in favor of (athlete/NADO)
	Sanction appealed
	Outcome (full sanction/reduced sanction/no sanction)
Sample and Procedures	In/Out-of Competition
	Date of Sample Collection
	Date of A sample
	Date of Notice
	Date of B Sample
	Date of Notice
	Date of Notification of Panel Hearing
Panel Procedures	Suspension start date (incl. provisional)
	No. of Panel Hearings
	Hearing Dates
	Hearing via teleconference (Yes/No)
	Date of Final Panel Hearing
	Days between first hearing and Panel Decision
	Days between Notification and Panel Decision
	Days between Sample and Panel Decision
ADAP Procedure	Appeal (Yes/No)
	Date of Appeal
	Number of ADAP hearings
	Hearing dates
	Date of Decision
	Outcome
	Days between appeal and Decision
CAS Appeal	Appeal (Yes/No)
	Date of filing of appeal to CAS
	Date of CAS Hearing
	Date of CAS Decision
	Days between appeal and Decision
	Outcome
Total days (Sample to Final Decision)	
Summary	

The selection of independent variables which focus on time lapsed in each phase of the disputes allows comparison between the actual duration of proceedings with: (i) the prescribed time limits under the Code and NADA Rules to determine compliance with the applicable law; and (ii) proceedings in other jurisdictions to determine the relative delay across jurisdictions. In addition, the collection of data with respect to whether an athlete is represented by a counsel in the proceedings will allow comparisons to be made across the three jurisdictions; India, New Zealand and Canada.

While the above data collection was substantially quantitative in nature, the summary also captured key qualitative information that would be useful in explaining any delays in proceedings, socio-economic constraints of the athlete which were argued in proceedings and other comments on procedural irregularities discussed in the cases, if applicable. Several other factors can have a significant impact on how an athlete experiences anti-doping rules and procedures, and many of these variables could not be coded given the absence of information in the available data. Such variables include whether the athlete spoke English, their age, level of education, where they lived, and the quality of their legal representation.

### Comparative analysis

A comparative analysis across jurisdictions will allow the researchers to determine whether any procedural irregularities are unique to India. As such, data have also been collected and coded from first instance disputes in Canada and New Zealand.

While awards are not publicly available in India, some jurisdictions have created publicly available databases, allowing people to access decisions and review jurisprudence of domestic decisions. The full awards from all domestic anti-doping cases in New Zealand and Canada which have been made publicly available were analyzed and coded. Both the Sport Dispute Resolution Centre of Canada (SDRCC)^70^ and the Sports Tribunal of New Zealand^71^ websites enable a search of their jurisprudence database, permitting filters for the type of dispute (doping) and dates of decision.

New Zealand and Canada were selected as useful comparative jurisdictions given the relative strength of sporting institutional infrastructure in each country as compared to India, and all jurisdictions are members of similar major events including the Olympic Games and Commonwealth Games. Importantly, both nations are considered developed economies, in contrast to India. In addition, both jurisdictions have evaluated their domestic doping dispute resolution systems and implemented reforms to streamline procedures.^72^ Most importantly, however, the institutions in New Zealand and Canada have published the majority of the full awards online, allowing access to similar data points for each jurisdiction as those collected for India.

Almost all anti-doping disputes were heard by the domestic panels at first instance across these three jurisdictions.^73^ In Canada, the majority of sports federations have adopted the Canadian Anti-Doping Program and therefore disputes are resolved through the Canadian Centre for Ethics in Sport (CCES) and administered by the Sport Dispute Resolution Centre of Canada. However, during the Data Collection Period, several cases were resolved through private arbitrations held by the international governing bodies, and a number of these were eventually appealed to CAS.^74^ These cases are outside the scope of the current research, which aims to analyze the effectiveness of the domestic dispute resolution processes at first instance. Measuring the procedural safeguards at first instance is crucial, given that the vast majority of athletes across all jurisdictions have their cases determined by domestic panels, and more often than not these decisions are not appealed to the CAS.^75^

### Analysis

In addition to the frequency analysis conducted on key variables, a chi-squared analysis of statistical independence was conducted among the frequency data across coded categories to test the statistically significant differences between key variables such as jurisdiction and delay, and legal representation and the outcome of the dispute.

## Results and analysis

### Timeliness and delay

#### India

In India, the average time between the date of the athlete’s sample collection to the date of the panel decision was 235.5 days (and 262 days, including any appeals). The vast majority of cases (96.67 percent) took more than three months to resolve from the date of sample collection, and a significant amount (9.83 percent) took more than one year to resolve from the date of sample collection. From the date the athlete was notified of the ADRV, 172 cases took more than three months to resolve at first instance (28.96 percent), 51 (8.59 percent) cases took more than six months to resolve, and 23 cases took more than one year to resolve (3.87 percent).

#### New Zealand

In New Zealand, the average time for these cases between sample collection and panel decision was 142 days (and 149 days, including any appeals). 27 cases (61.36 percent) required more than three months to be resolved, whereas only two cases (4.55 percent) required more than one year to be resolved from the date of sample collection.^76^ No data were available on the date of notification of the ADRV.

#### Canada

In Canada, the average time for cases between sample and panel decision was 176 days (and 180 days, including any appeals). 31 cases (83.78 percent) of cases required more than three months to be resolved, whereas only three cases (8.11 percent) of cases required more than one year to be resolved from the date of sample collection. 16 cases (43.24 percent) of cases required more than three months to be resolved, whereas no cases required more than one year to be resolved from the date of notification to the athlete of the ADRV.

**Fig. 1:**
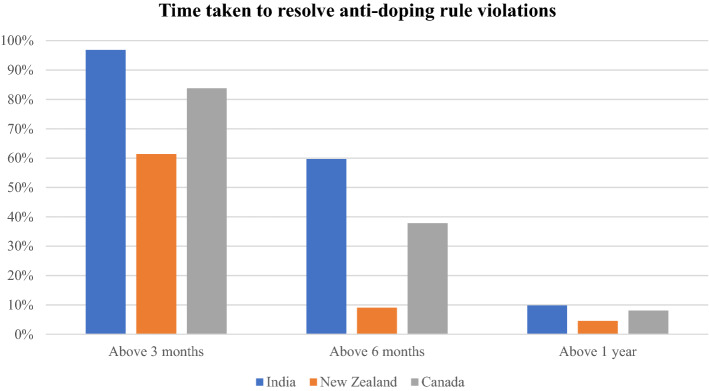
Comparison of time period which anti-doping disputes are resolved in India, Canada and New Zealand

A comparison of the length of time taken to resolve these disputes is set out in Table 2 and details of the time period in which anti-doping disputes are resolved across the three jurisdictions are set out in Table 3. Figure 1 illustrates the time periods (3 months, 6 months, or 12 months) within which cases have been resolved in each jurisdiction. It is evident from the data that Indian anti-doping cases take longer than similar cases in Canada and New Zealand.

**Table 2:** Comparison of length of time taken to resolve anti-doping disputes in India, Canada and New Zealand

Country	Number of cases	Average time between Sample and Panel Decision (in days)	Median time between Sample and Panel Decision (in days)	Average time between Sample and Final Decision (including appeals) (in days)
India	594	235.50	200	262
New Zealand	43	142	114	149
Canada	37	176	152	182

**Table 3:** Comparison of time period in which anti-doping disputes are resolved in India, Canada and New Zealand

	Sample to decision			Notification to decision		
	Above 3 months (%)	Above 6 months (%)	Above 1 year (%)	Above 3 months (%)	Above 6 months (%)	Above 1 year (%)
India	96.83	59.67	9.83	28.50	8.50	3.83
New Zealand	61.36	9.09	4.55	NA^a^	NA	NA
Canada	83.78	37.84	8.11	43.24	16.22	0.00

^a^Note that date of notification is not available from New Zealand awards

### Compliance with time limits

In India, under the 2010 NADA Rules, which are applicable to 95.4 percent of cases in the sample, anti-doping panels were required to meet strict timelines,^77^ In particular, the ADDP was required to:

(i) commence the hearing within 14 days of the notification date;^78^
(ii) issue a written decision within 20 days of the notification date;^79^
(iii) issue written reasons for the decision within 30 days of the notification date,^80^ and
(iv) hearings should be completed within three months of the conclusion of the results management process.^81^

By contrasting these time limits with the actual time taken by the ADDP, the researchers determined whether the ADDP complied with the requisite procedural requirements in applicable rules. As illustrated in Table 4, the findings show that:^82^

(i) A total of 45 cases (7.95 percent) commenced the hearing within 14 days of the notification date.
(ii) A total of 5 cases (0.88 percent) issued a written decision within 20 days of the notification date.
(iii) A total of 30 cases (5.3 percent) issued written reasons for the decision within 30 days of the notification date.
(iv) A total of 172 cases (30.39 percent) were completed within 3 months of the conclusion of the results management process.

**Table 4:** Compliance of the anti-doping dispute panel with time limits prescribed under the NADA rules

Applicable law	Compliant requirement	Compliant		Not-compliant		Not available	
2010 NADA rules	Commenced the hearing within 14 days of notification date	45 cases	7.95%	511 cases	90.28%	10 cases	1.77%
	Issued written decision within 20 days of notification date	5 cases	0.88%	551 cases	97.35%	10 cases	1.77%
	Issued written reasons for the decision within 30 days of notification date	30 cases	5.30%	526 cases	92.93%	10 cases	1.77%
	Hearings should be complete within 3 months of the completion of the results management process	172 cases	30.39%	384 cases	67.84%	10 cases	1.77%
2015 NADA rules	Commence within 45 days of the constitution of the panel	24 cases	85.71%	4 cases	14.29%	NA	NA
	Issue written decision with its reasoning within 90 days of the constitution of the panel	5 cases	100%	NA	NA	NA	NA
	Hearings completed within 3 months of the completion of the results management process	28 cases	100%	NA	NA	NA	NA

It is interesting to note that the 2015 NADA Rules relaxed the strict time limits and as a consequence, the ADDP’s compliance has improved. Under the 2015 NADA Rules, the anti-doping disciplinary panel was required to (i) “commence … within 45 days of the constitution of the … panel”, (ii) “issue a written decision with its reasoning within 90 days of the constitution of the … panel”,^83^ and (iii) hearings “should be completed … within three (3) months of the completion of the results management process …”.^84^

The 2015 NADA Rules were only applicable to 28 cases in the Data Collection Period. For these cases, the compliance with the relaxed rules was much more consistent. As illustrated in Table 4, 24 cases (85.71 percent of cases) complied with the first requirement, while all 28 cases (100 percent of cases) complied with the second and third requirements.

Similar comparisons could not be made for New Zealand and Canada due to the lack of data available for key variables such as the date that the athlete was sent the notice of charge.

The systemic non-compliance, under the 2010 NADA Rules in particular, is consistent with the researcher’s hypothesis that there is a gap between the question of harmonization in the anti-doping rules and the enforcement of those rules. However, despite the small sample of cases since the 2015 amendments to the NADA Rules, there appears to have been a trend towards faster resolution of disputes given that all hearings were completed within the required three months from the conclusion of the results management process.

### Use of technology in dispute resolution

In all three jurisdictions, technology (such as telephones or videoconferencing) is permitted to be used for anti-doping hearings and pre-hearings. From a procedural perspective, the use of such technology can improve timeliness (especially with respect to scheduling hearings between counsel and arbitrators since travel is no longer a requirement), and access to justice, especially in a jurisdiction such as India since athletes may otherwise be required to travel from remote areas to the tribunal in Delhi for a physical hearing.

In New Zealand, of the 43 cases in the sample, 35 hearings were conducted via telephone, 7 in person, and one did not have a hearing. In Canada, all disputes commenced with a pre-hearing procedure, following which a total of 11 of the 37 disputes had full hearings via teleconference. In contrast, no hearings in India were recorded as having been conducted via telephone or videoconferencing during the Data Collection Period. As discussed below, the fact that Indian tribunals have not used technology during the hearing process could have implications on procedural fairness (including access to justice and timeliness) and could be one justification for the more timely resolution of disputes in New Zealand and Canada.

### Access to justice and legal representation

Only 4.88 percent of athletes were represented by legal counsel at first instance hearings in India. It follows that 95.12 percent of athletes were either self-represented, or appeared alongside a coach, relative, or friend during the hearing. A higher proportion of athletes were represented by counsel on appeal than at first instance hearings. Of the 56 athletes who appealed their first instance decisions to the ADAP, 53.57 percent were represented by counsel.

**Fig. 2:**
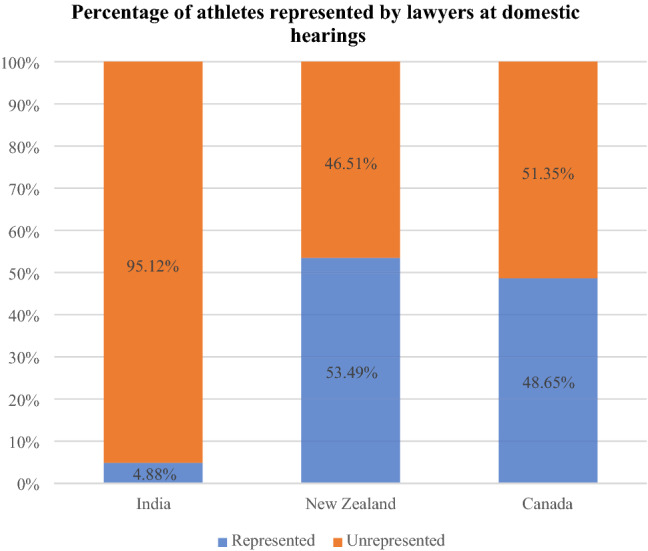
Percentage of athletes represented by legal counsel in first instance anti-doping panels

There is a significant difference between the number of athletes who are represented by legal counsel at first instance hearings in India (4.88 percent) as opposed to New Zealand (53.49 percent) and Canada (46.51 percent). This suggests potential access to justice barriers in India. While the summary of these different levels of legal representation is set out in Table 5, the visualization in Fig. 2 highlights the stark difference between legal representation at first instance anti-doping panels in India, when compared to the other two jurisdictions.

**Table 5:** Number of athletes represented by legal counsel in domestic anti-doping panels

Country	Panel	Sample size	Number of athletes represented by lawyers	Number of athletes not represented by lawyers
India	Anti-Doping Disciplinary Panel	594	29 (4.88%)	565 (95.12%)
	Anti-Doping Appeal Panel	56	30 (53.57%)	24 (42.86%)
New Zealand	Sports Tribunal of New Zealand	43	23 (53.49%)	20 (46.51%)
Canada	Sport Dispute Resolution Centre of Canada	37	18 (46.51%)	19 (51.35%)

### Legal representation and hearing outcomes

**Fig. 3:**
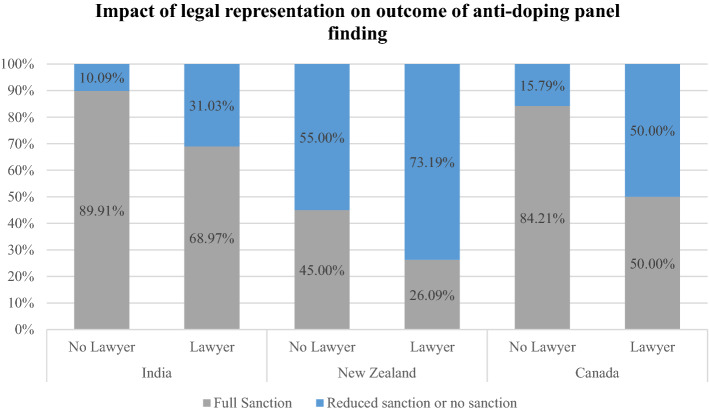
Impact of legal representation on the outcome of anti-doping panel finding at first instance

Given this disparity in legal representation, we analyzed hearing outcomes, specifically whether athletes received a full sanction under the Code (i.e., the maximum of 2 years) or whether their sanction was either partially reduced (for instance, from a maximum sanction of two years to a reduced sanction of 1 year) or completely waived. The authors hypothesized that legal representation may have some bearing on whether the athletes’ sanctions would likely be reduced or waived. Across all jurisdictions, the data show that when an athlete is represented by a lawyer, they are more likely to receive a more favorable outcome. This correlation is summarized in Table 6 and illustrated in Fig. 3.

**Table 6:** Impact of legal representation on the outcome of anti-doping panel finding at first instance

Country	Sanction	Total cases	No lawyer		Lawyer		Percentage difference
India	Full sanction	528	508	89.91%	20	68.97%	20.94%
	Reduced sanction	66	57	10.09%	9	31.03%	
New Zealand	Full sanction	15	9	45.00%	6	26.09%	18.19%
	Reduced sanction	28	11	55.00%	17	73.19%	
Canada	Full sanction	25	16	84.21%	9	50.00%	34.21%
	Reduced sanction	12	3	15.79%	9	50.00%

In India, if an athlete was represented by a lawyer there was a 31 percent chance that they would receive a reduced sanction, whereas there was a 10 percent chance that they would receive a reduced sanction if they were unrepresented. Accordingly, athletes were 21 percent more likely to receive a more favorable outcome if they were represented by legal counsel at first instance. A chi-square test of independence was performed to examine the relationship between reduced or no sanction and having a lawyer versus not having a lawyer in India. The relationship between these variables was significant in India, *X*^*2*^ (1, *N* = 29) = 14.02, *p* = 0.00018, indicating that there is a statistical difference in outcome between having a lawyer versus not having a lawyer in India.

In New Zealand, 73.19 percent of athletes who were represented by a lawyer received a reduced sanction, whereas 55 percent received a reduced sanction if they were unrepresented. Accordingly, athletes were 18.19 percent more likely to receive a more favourable outcome if they were represented by legal counsel at first instance. The relationship between these variables was not significant, *X*^*2*^ (1, *N* = 23) = 3.32, *p* = 0.06827, indicating that there is not a statistical difference between having a lawyer versus not having a lawyer and outcome in New Zealand.

In Canada, 50 percent of athletes who were represented by a lawyer received a reduced sanction, whereas 15.79 percent received a reduced sanction if they were unrepresented. Accordingly, athletes were 34.21 percent more likely to receive a more favourable outcome if they were represented by legal counsel at first instance. The relationship between these variables was significant, *X*^*2*^ (1, *N* = 18) = 15.84, *p* = 0.000069, indicating differing outcomes between having a lawyer versus not having a lawyer in Canada.

### Legal representation and timeliness

While there is some evidence to suggest that cases involving lawyers took longer in India and Canada (see Table 7), there is insufficient information to draw any conclusions with respect to New Zealand cases (because no data on the date of notification of ADRV is available). Since lawyers are typically only involved after the date of notification of the ADRV to the athlete, this is the relevant data point to consider when analysing whether lawyers have had any impact on the time taken to resolve disputes.

**Table 7:** Time taken to resolve an anti-doping violation charge vs whether an athlete is represented by a legal counsel

	Time taken (notification to decision)	
	Lawyer	No lawyer
India	420.57	87.52
New Zealand	NA	NA
Canada	155.33	97.79

## Discussion

### Timeliness

#### Context

Time is of the essence for athletes during anti-doping proceedings for a number of reasons. First, a matter of months or years can be career ending from an athlete’s perspective, especially given the relatively short period of time that elite athletes can perform at their peak.^85^ To this end, undue delay in anti-doping proceedings can result in an athlete serving a longer sanction than that ultimately handed down by the panel,^86^ leading to irreparable harm to the athlete’s career. In addition, procedural delays can result in substantive unfairness to the athlete, especially where such delay leads to difficulties in bringing certain types of evidence before the panel. It has, for example, previously been argued that delay in notification to an athlete of an adverse analytical finding may result in the athlete no longer being able to prove the source of the prohibited substance (that is, how the substance entered into their body).^87^ Due to the importance of timeliness, strict time limits have been imposed by the Code and various national regulations, including the NADA Rules. The most recent amendments to the Code in 2021 provide timeliness as a guiding principle. In particular, the ISRM provides that

*In the interest of fair and effective sport justice, antidoping rule violations should be prosecuted in a timely manner. … Anti-Doping Organizations should be able to conclude Results Management (including the Hearing Process at first instance) within six (6) months from the notification [of the ADRV to the athlete].*^88^

#### India

Despite the importance of timeliness in anti-doping disputes, there have been systemic delays in anti-doping disputes in some jurisdictions, including in India. In India, cases required an average of 235.5 days to be resolved from the time of sample collection. 97 percent of cases required more than 3 months to resolve, and 10 percent of cases required more than 1 year to resolve. Even using the new ISRM timeliness mandates as a benchmark, 8.59 percent of Indian cases required more than six months to be resolved from the date of notification of the athlete. In fact, the longest case in India required more than 1000 days to be resolved from the sample collection to the decision by the first instance panel.^89^ There was no explanation provided within the award of this case for the extended delay, including the 1.5-year delay between the notice of the athlete’s B sample to the constitution of the panel. While this case is an outlier, there were 20 cases that required two years or more to resolve from the date of sample collection.

While there does not appear to be a correlation between the number of ADRVs and the time taken to resolve cases, further empirical research would be beneficial to understand why delays were considerably longer in 2011 and 2012 (276 and 290 days respectively) than 2013 and 2014 (204 and 167 days, respectively). The number of and relative experience of arbitrators may intuitively impact the timeliness of dispute resolution as one would logically assume that a larger number of arbitrators can dispose of a higher number of cases and that more experienced arbitrators can resolve complex procedural issues more efficiently. However, researchers have argued that this is not necessarily the case in civil disputes.^90^ Accordingly, it would be valuable to calculate the impact of the number of arbitrators listed during these years, and the relative experience of these arbitrators. In addition, insights into how case management approaches differ between different jurisdictions would be valuable. However, such data is not publicly available. In any event, further capacity building and training of arbitrators about case management and the importance of time limits in anti-doping disputes would be valuable in the Indian context.

Ostensibly, compliance with the 2015 NADA Rules improved significantly when compared to compliance with the time limits under the 2010 NADA Rules. However, the time limits imposed on panels were relaxed significantly under the 2015 version of the rules, to be more consistent with the procedural standards under the Code. Therefore, while compliance did improve, there is no evidence of structural or policy changes in India to incentivize more efficient procedures. To further understand the reasons for non-compliance with the prescribed time limits under the NADA Rules, further primary research could be conducted in the form of surveys or interviews of former arbitrators of the ADDP, or legal counsel involved in hearings during the Data Collection Period.

#### Comparative discussion

Consistent with the hypothesis that there is a lack of harmonization in the implementation of anti-doping procedures between developed and developing countries, first instance anti-doping cases take longer to be resolved in India than in Canada and New Zealand. While almost 60 percent of cases took longer than six months to resolve in India from the date of sample collection, in Canada (37.8 percent) and New Zealand (9 percent) far fewer cases required more than six months to reach a final decision on an athlete’s ADRV. There are a number of possible reasons for the extent of the delay in India as opposed to the relatively speedy dispute resolution systems of the other countries. Further empirical research is required to understand what causes these delays in India, as such research could inform positive reform in India’s dispute resolution process.

##### Strict procedural timelines and compliance

The Code and the regulatory rules of each of the jurisdictions studied emphasize the importance of timeliness in the results management process and the panel hearings. However, compliance with these timelines varies across jurisdictions. Under the applicable rules in New Zealand, the importance of timeliness is at the heart of the dispute resolution process. The Rules of the Sports Tribunal of New Zealand expressly provide for a “just, speedy and inexpensive determination of any proceeding” and further provide that the tribunal has the power to make orders “… as it considers to be consistent with the just, speedy and inexpensive determination of the Proceeding.”^91^ David has noted that the tribunal has generally delivered on their aim to produce reasoned decisions in a timely and cost-effective manner, and the data in this study reflects this.^92^ Conversely, as discussed above, the NADA Rules in India relaxed the previously strict time limits in 2015, presumably due to the high non-compliance in the vast majority of cases under the previous versions of the rules.

Revisions under the 2021 version of the Code have further enshrined these timelines as mandatory procedural guarantees.^93^ However, as commentators have noted, it is important that WADA, the CAS and NADOs properly implement the existing strict timelines to ensure efficiency and fairness in the process.^94^ While sanctions for non-compliance may be one solution, the importance of education and capacity building is critical, especially amongst NADOs in developing countries. Accordingly, the appointment and training of tribunal members are critical in ensuring that the integrity and efficiency of the dispute resolution process are maintained.^95^ It can be argued that better trained and more experienced arbitrators can identify and resolve complex substantive and procedural issues more efficiently.

##### Local legal culture and delay

It is important to note that while timelines and other procedural safeguards are ostensibly harmonized under the Code, the implementation of such safeguards will inevitably vary given the different cultural, legal, economic and institutional contexts within each jurisdiction.^96^ Compared to New Zealand and Canada, India has a much larger population, and its dispute resolution culture is notorious for its slow litigation procedures, within the context of both civil and criminal law.^97^ Galanter and Krishnan (2004) argued that Indian courts and tribunals are “beset with massive problems of delay, cost, and ineffectiveness”.^98^ As such, the relative delay in proceedings in anti-doping disputes cannot be viewed in isolation. Scholars have argued that a cultural paradigm shift is required to ensure speedy dispute resolution in India,^99^ and this same argument can be extended to anti-doping procedures. However, regardless of local legal culture, WADA’s quest for a harmonized approach to anti-doping requires it (as well as the respective NADOs) to promote timeliness, justice and procedural fairness irrespective of jurisdiction.

##### The role of technology in dispute resolution

Perhaps one of the reasons for New Zealand’s efficiency and speedy process, as compared to India for example, is the accessibility and use of technology. In New Zealand, the use of technology (such as teleconference or videoconference) is accepted practice.^100^ David (2016) notes that this practice has generally worked well and that

*… this method of hearing proceedings has been driven primarily by the logistical difficulties in arranging urgent hearings involving parties from around New Zealand and the considerable cost savings for all parties and, in particular, athletes.*^101^

Under the applicable law, New Zealand permits hearings (and pre-hearings) via telephone to ensure a speedy dispute resolution procedure.^102^ In practice, 81.4 percent of cases conducted hearings via telephone. In Canada, the doping panel is required to “convene a preliminary meeting of all parties by teleconference to settle procedural matters”.^103^ The full oral hearing may be conducted by video or teleconference.^104^ In practice, a total of 29.7 percent of disputes had full hearings via teleconference in Canada.

This is in stark contrast to hearings in India where no cases were recorded as having telephone hearings, despite panels having discretion under the NADA Rules to permit telephone and video conferencing to be used for parties to present evidence, including the right to call and question witnesses.^105^ However, in more recent times, in particular, during the COVID-19 pandemic, proceedings have been conducted using technology across jurisdictions, including in India. The move to completely online hearings was experienced globally during the pandemic, with some jurisdictions being able to adopt more quickly as a result of prior practices and infrastructure.^106^

Any policies that incorporate technology into dispute resolution systems, however, need to acknowledge the inequalities with respect to access to technology, especially for athletes in rural areas. As such, traditional hearing options, as well as technology hubs where virtual hearings could take place in closer proximity to such athletes ought to be considered among any reform measures. Other procedural rights, such as the athlete’s right to an interpreter (under Article 8 of the Code), must continue to be respected in online hearings, especially given the language barriers that exist in multilingual countries such as India.

##### Scale: number of anti-doping rule violations (ADRVs) and efficiency of panels

Traditional theories of courtroom delay focus on “large caseloads thrust upon mismanaged and inefficient courts”.^107^ While conventional wisdom suggests that delay is more complex than just large caseloads, it is logical that judges or arbitrators with a high volume of cases may *prima facie* take longer to resolve them on average. During the Data Collection Period, there were far more anti-doping cases before first instance panels in India (594), than in Canada (37) and New Zealand (43). Indeed, India is consistently ranked as one of the worst doping offenders in the world, according to WADA reports.^108^ Information with respect to the number of panel members who were appointed by each of the domestic first instance panels during the Data Collection Period is not publicly available. Further research to compare the number of arbitrators in each jurisdiction, the number of cases heard by each arbitrator and their overall capacity to hear cases would assist policymakers in better understanding the impact that the caseload of each arbitrator has on delay of cases in the respective jurisdictions.

##### Role of lawyers and delay

In India, cases in which a legal representative was present required on average 333 days longer to resolve than cases where athletes were unrepresented, whereas in Canada cases required an average of 57 additional days to resolve where an athlete was represented by a lawyer. While this seems to be a significant trend, there is again likely to be an inherent bias in this data given that more complicated cases, where athletes wish to adduce evidence of their innocence, may require much longer than cases where an athlete concedes guilt. Similar questions of the impact of lawyers on dispute resolution systems have been examined by scholars previously, with Lederman and Hrung (2006) noting that while the presence of a lawyer increased the time to trial, it had “no significant effect on the time elapsed between filing and trial”.^109^ Further empirical research would be valuable to examine why cases involving counsel required longer to resolve, and which stages of the dispute resolution process took longer. Qualitative research could be conducted by coding the full-text judgments to understand the types of evidence (and arguments) brought by the athletes in cases where they were represented by counsel, and whether this differed from unrepresented litigants. For instance, coding these awards to ascertain whether an athlete concedes guilt, or argues their innocence, or that they inadvertently consumed a prohibited substance, to examine whether there is any association between perceptions of guilt with legal representation and delay. Since cases involving alleged inadvertent doping present a whole new level of evidential complexity, with an onus of proof on the athlete, we hypothesize that such cases will inevitably take longer to resolve.

### Access to justice

While the Code requires that athletes are provided access to legal representation at their own cost, it is clear that a significant majority of athletes in India, and a large minority of athletes in Canada and New Zealand did not have legal representation for their anti-doping proceedings. As a result, many domestic anti-doping panels around the world have introduced pro bono panels or financial aid for athletes.^110^ The same is true for the CAS. While in India, the NADA Rules provide that each party has the right to be represented at a hearing at their own expense,^111^ there is no additional financial aid or institutional infrastructure to support or promote a list of pro bono lawyers, as has been the case in other jurisdictions.^112^ As a consequence, many athletes are without counsel when they appear before the anti-doping tribunal in India.

Despite the dearth of literature on the importance and the impact of legal representation for athletes in anti-doping disputes, empirical research does exist on the impact of lawyers in civil disputes. As discussed above, research suggests that access to lawyers significantly increases the chances of success in civil courts^113^ and other types of disputes.^114^ This is logical, given the complexities of navigating the justice system,^115^ and as a consequence, research suggests that self-represented litigants often make “elementary errors”.^116^ Not only are counsel (particularly experienced counsel) likely to have better knowledge of the procedural nuances of the dispute resolution system, but they are also likely to find and better argue arguments in favor of their clients. This is consistent with a study of the Wisconsin Tax Appeals Commission which found that lawyers succeeded in reversing the Department of Revenue’s determination in 36 percent of cases, while unrepresented taxpayers were similarly successful in only 20 percent of the cases.^117^ Another study of decisions of employment arbitrations before the American Arbitration Association showed that an employee represented by a lawyer succeeded in 22.9 percent of cases, whereas self-represented employees succeeded only 18.3 percent of the time.^118^ These studies confirm the theory that the presence of counsel has a significant impact on the final outcome of such cases. In fact, a review of the empirical studies on the impact of legal representation suggests that “… lawyer-represented focal parties are more than 5-times more likely to prevail in adjudication than self-represented litigants, and 40% more likely to prevail than parties represented by non-lawyer advocates”.^119^ Studies also suggest that “the complexity of the law and procedure involved play significant roles in shaping lawyers’ impact on how cases turn out”.^120^ The anti-doping framework is complicated. The procedural nuances and scientific complexities which are required to interpret testing laboratory reports, for example, are almost prerequisites to adequately defend an alleged ADRV in an anti-doping dispute. Accordingly, it follows that legal representation is likely to have a significant impact on the outcome of anti-doping proceedings. This was reflected in the data which showed that athletes were significantly more likely to receive a favorable outcome if they were represented by a lawyer in anti-doping disputes, across all three jurisdictions. This is particularly concerning in India given the large number of unrepresented athletes and the lower levels of literacy when compared to the other jurisdictions. These findings have clear policy implications, especially with respect to the procedural rights of athletes. It follows that if legal representation of athletes is not improved in India, it is even more important that athletes are educated about their procedural rights and provided direct assistance by tribunal members throughout the process. NADOs and federations have a responsibility to create and implement education programs which ensure that athletes are aware *inter alia* of their rights and responsibilities under the Code.^121^ There is little evidence to suggest that this is taking place in India, and further research is required to assess athletes’ knowledge of their procedural rights under the Code.

As a result of an imbalance of resources and experience, Galanter (1974) suggests that the stronger party is most likely to prevail in a dispute, and as such institutional litigants often succeed more than individuals.^122^ This is particularly true in anti-doping where the WADA and NADOs are “repeat players”, whereas athletes are “one-shotters” and therefore inevitably have less experience in navigating the dispute resolution system.^123^ It is, therefore, unsurprising that athletes who are represented by legal counsel are more likely to succeed than self-represented athletes with no experience in the system. This is also consistent with empirical research conducted on taxation disputes which suggests that “… some combination of attorneys’ greater expertise, experience, and familiarity with the Tax Court and its judges improves the outcome for the taxpayer”.^124^ Further research into the impact of a legal counsel’s experience in anti-doping on the impact of the outcome of the sanction would be a worthwhile addition to the future research agenda. While it is expected that experience and outcomes are positively correlated, no empirical evidence has established this in an anti-doping context to date.

Further analysis is required to ascertain whether athletes choose to be self-represented across all countries, or whether their lack of legal representation is due to institutional shortcomings. The relatively lower levels of legal representation in anti-doping disputes in India may also be a reflection of the legal culture and access to justice barriers that exist in each of these countries, even outside of anti-doping, with high costs of legal representation evident in India despite the relatively lower socio-economic profile of many respondents.^125^ Conversely, legal reform in Canada and New Zealand in recent years has led to the vast majority of athletes being represented by lawyers. Through institutional reforms such as the creation of pro bono counsel lists or legal aid options for athletes,^126^ athletes who have been accused of an ADRV in New Zealand and Canada now have far greater access to lawyers. As a consequence, if this study were to be repeated using data from the past three years, the percentage of athletes represented by counsel will be significantly higher. Such reforms which have been adopted by the CAS as well as other developed countries have led to an increase in legal representation in anti-doping disputes. Indeed, data from Sport Resolutions UK suggests that since 2017, more than 96 percent of athletes have been represented, with more than 60 percent of all athletes having been represented by counsel on a pro bono basis.^127^ However, similar reforms which adopt pro bono lists or legal aid funding have not been adopted in India and as such it is likely that the majority of athletes continue to be self-represented in anti-doping disputes. While access to anti-doping awards in India are not publicly available, if access was to be provided by the Ministry a longitudinal analysis would be valuable to ascertain whether access to legal counsel has improved in recent years.

While it is logical to assume that given the complexity of anti-doping law, athletes would benefit from legal representation in such disputes, there is no previous empirical evidence to date that suggests that legal representation will result in a more favorable outcome. However, according to the data in this study, an athlete is more likely to receive a more favorable outcome (a reduced sanction or no sanction at all) if they are represented by a lawyer, regardless of the jurisdiction of the dispute. However, as has been noted by scholars assessing the impact of legal representation in employment disputes, “… there is likely to be a selection effect in which counsel can identify in advance cases where the employee is more or less likely to be successful”.^128^ Similarly, athletes are more likely to engage a counsel where they believe they have a higher chance of receiving a reduced sanction. Therefore, similar to Colvin’s (2011) study of employment arbitration, “[t]he cases in which employees do have representation by counsel are on average those in which they have a greater chance of success...”^129^ The association between legal representation and a favorable outcome, while significant, shows a correlation, rather than causation. However, given that athletes were 20 percent more likely to receive a favorable outcome if they were represented by a lawyer in Indian anti-doping disputes (and similarly 18.19 percent and 34.21 percent more likely in New Zealand and Canada, respectively), further research is warranted. Interestingly, while there is a statistically significant association between legal representation and hearing outcome in India and Canada, there is not a statistical difference between having legal representation and hearing outcome in New Zealand. This is perhaps due to the fact that a large proportion of athletes who are self-represented are still awarded a reduced sanction at first instance in New Zealand (55 percent), which is not the case in India (10.09 percent) and Canada (15.79 percent). Therefore, while athletes in New Zealand are still 18 percent more likely to receive a more favorable outcome if they are represented by a lawyer, the relative impact of a legal representation is less than in the other jurisdictions. This may be because of “procedural rules which are simple … and can be flexibly applied” in favor of justice and efficiency, as well as a tribunal composed of “a significant number of experienced lawyer members”.^130^

In any event, policy reforms which have taken place in developed countries that promote legal representation of athletes enhance the legitimacy of the anti-doping system. If athletes have access to, and can afford, legal representation in anti-doping disputes, they are more likely to have their procedural rights protected. As set out in WADA’s Athlete’s Anti-Doping Rights Act, it is recommended that all “Athletes should have the right to access legal aid for hearings and appeal process in doping cases”.^131^ However, it should be acknowledged that while resource constraints may prevent some countries from adopting the highest of standards in anti-doping procedure,^132^ such as financial aid for accused athletes, there are numerous practical measures that NADOs and domestic panels can adopt to ensure that all athletes have access to legal representation. For instance, the creation of institutionalized pro bono lists of lawyers and awareness programs would be of immense value for accused athletes and a minimal cost to resource-poor NADOs and panels.

Further research would provide a more granular understanding of why the association between legal representation and reduced sanction is occurring and would offer further evidence to inform policy changes. One approach would be to survey athletes or athletes’ counsel on their perspectives of the anti-doping process to calibrate the “hard data” from this study. Athletes’ counsel can be a useful resource in designing future research because while athletes are typically “one shotters” in anti-doping disputes, there are numerous lawyers with significant experience in anti-doping matters and they can play an important role in informing a more balanced debate on understanding whether athletes’ rights are adequately protected in anti-doping disputes as well as proposing appropriate recommendations for procedural reform. Interviews with NADOs and first instance panel members are also likely to provide rich qualitative data on why challenges of delay and access to counsel exist. Such data would be useful in informing our understanding of the unique nuances of different legal cultures and provide a valuable tool for promoting institutional reform across jurisdictions. Another research approach could adopt a more thorough content analysis of the awards published by panels, as this would help researchers ascertain whether an athlete claims that they unintentionally or unknowingly consumed the prohibited substance – this would allow researchers to code which cases an athlete has attempted to put forward a “defense” to seek a reduced sanction, and whether representation by a lawyer has impacted the outcome of these disputes in particular. While outside the scope of this paper, such research would be a valuable contribution to the question of access to justice in anti-doping disputes.

### Limitations

There are a number of limitations to this study. Since NADA does not publish cases handed down by domestic panels, the authors have relied on the Ministry of Youth Affairs and Sport for access to the awards. Despite requests from the researchers, access to cases handed down after August 2016 has not been provided by the Ministry due to administrative and resource constraints. Although the central tenets of procedural fairness have remained largely unchanged under the Code until the recent amendments in 2021, only limited data were available since these amendments came into force on 1 January 2021. To this end, when further data become available, further empirical research would be beneficial. Such research may take the form of a longitudinal study that analyzes the impact of the amendments to the minimum procedural guarantees enshrined under the 2021 Code and the ISRM, in comparison with the earlier (less stringent) protections under the earlier versions of the Code. In addition, access to cases wherein an athlete has successfully defended their ADRV have not been shared by the Ministry on the grounds of confidentiality. The limited availability of the case law, while an issue of access to justice and transparency in itself,^133^ is an inevitable challenge in data collection in anti-doping disputes across most jurisdictions. Indeed, the challenge of empirical research with respect to arbitral awards is not unique to anti-doping disputes. Lindholm (2019) noted that “[t]he confidentiality of the proceedings and the awards is a major methodological challenge when studying arbitration tribunals, particularly when conducting quantitative research that requires a representative data sample”.^134^ However, as discussed above, the sample still equates to 95 percent of all anti-doping violation cases in India during the Data Collection Period. Despite these limitations and as a result of the absence of full-text awards in the public domain, the analysis of more than 600 awards across three jurisdictions contributes significantly to our understanding of compliance with procedural safeguards at first instance tribunals.

Another limitation is the lack of access to proceedings of the disputes and the briefs of the parties.^135^ An additional study that surveys athletes’ lawyers and representatives, may be able to fill this void, and while outside the scope of this paper, this project is proposed in the future research agenda. The researchers observed during the coding process that there were eleven awards with incomplete data due to incomplete scanning or missing dates due to clerical errors by NADA. To this end, the researchers note that one limitation of this study is that it relies on the panels accurately recording whether the athlete was represented by counsel.

Finally, an analysis of other sporting jurisdictions such as Australia, the United States of America, Russia, China and the United Kingdom would have been useful from a comparative perspective. However, these jurisdictions have not published anti-doping awards online to the same extent as New Zealand and Canada.^136^ In addition, the authors acknowledge that the current data set is from common law countries, and as such, further research should focus on data collection from civil law jurisdictions. In particular, this study should be extended to European countries to analyze whether there are any consistent trends in first instance disputes and whether the jurisprudence of the European Court of Human Rights with respect to fair trial rights and procedural fairness has had an impact on such tribunals. Accordingly, it is acknowledged that further studies could be conducted with respect to these other jurisdictions in the future, should this data be made publicly available.

### Reform agenda

There is scope for reform to promote procedural fairness for athletes in anti-doping disputes, with a specific focus on access to legal representation and timeliness.

With respect to timeliness and delay, NADOs and first instance tribunals need to focus on strict implementation of the procedural safeguards which are now prescribed under the ISRM and the Code. WADA itself acknowledges that monitoring NADOs for compliance is a central responsibility of WADA to ensure harmonization so that “athletes know what to expect from the anti-doping system no matter where they are from or where they are competing”.^137^ However, sanctioning non-compliance with time limits is not the only option. Scholars have previously suggested that capacity-building programs may be much more effective. Müller (2017) suggests that NADOs could be required to cooperate with other NADOs to facilitate exchange programs and to enhance quality and harmonization.^138^ For instance, countries with a strong track record of timely dispute resolution could facilitate knowledge transfer and capacity-building programs in collaboration with developing countries, to encourage reforms which may reduce delays in proceedings. The Indian and Australian NADOs, for example, entered into a 2-year MOU in 2016 to “ensure India implements a more effective anti-doping program that is fully compliant with the [Code]”.^139^ Such agreements ought to be systematically promoted and monitored by WADA, and these collaborations should include capacity building for implementing best practices in case management. Under the NADA Rules, panel members of the ADDP are typically a mix of professionals from law, medicine and sport. This is generally consistent with the composition of other domestic anti-doping panels. Regardless of their professional background, members of such first instance panels may benefit from capacity-building programs, especially when procedural reforms and changes take place when the Code is revised (typically every 6 years). In addition, efficiency and timeliness should be central to proceedings, as is the case in New Zealand. The use of technology has been used effectively during the COVID-19 pandemic, across jurisdictions, and telephone and videoconferencing options should be available at the option of the parties to improve the efficiency of proceedings. Even prior to the pandemic, there have been shifts towards using electronic case management systems to improve efficiency and promote procedural fairness. Even the most “basic electronic case management systems” can enable courts and tribunals to “track cases, introduce process improvements based on facts, communicate better with other authorities and be better accountable to society”.^140^ As such, while some domestic anti-doping bodies have embraced technology more than others, jurisdictions such as India would benefit from electronic case management which would allow milestone planning, capacity allocation, workflow management, as well as tracking and tracing of cases.^141^ This would act as an early warning system to NADOs and independent panels whether (and at what stage) cases are being delayed and will allow domestic panels to make administrative decisions in real-time to improve the efficiency and effectiveness of their dispute resolution procedures.

With respect to access to legal representation, legal aid and pro bono counsel lists have proven effective in several countries.^142^ WADA acknowledges that “Athletes should have the right to access legal aid for hearings and appeal process in doping cases,”^143^ yet many jurisdictions have not adopted policies that support athletes when they are faced with an ADRV. In addition, there are various mechanisms whereby athletes could be made aware of pro bono lists and financial support, for instance through the creation of an athlete’s ombudsman,^144^ or through sharing the list of available support and pro bono counsel attached with the athlete’s notice of charge. As has been suggested by scholars, providing all litigants with lawyers “would be one way to level the playing field”.^145^ Some jurisdictions have gone as far as ensuring mandatory representation for accused athletes – for instance, in Brazil it is mandatory for any athlete accused of an ADRV who requires representation to be provided with a public defense attorney unless they choose to be unrepresented.^146^It should be noted that in October 2021, the WADA Athlete Commission proposed the establishment of an Athletes’ Anti-Doping Ombuds which would “establish a neutral or impartial dispute resolution practice whose major function will be to provide confidential and informal assistance to athletes bound by anti-doping rules under the World Anti-Doping Code”.^147^ WADA should implement this proposal, and in doing so create regional or domestic institutional ombudsmen to ensure that local and cultural nuances are respected. In addition, the simplification of procedures and other forms of institutional support would enable athletes to better navigate anti-doping procedures. Targeted institutional reform such as the establishment of a national sports tribunal in India might go some way to improving such procedures. The publication of awards should also be encouraged by national panels to promote transparency and accountability.

While it is not uncommon for WADA to revoke accreditation from testing laboratories for non-compliance with testing procedures and standards, there are often little or no consequences for national doping tribunals which fail to protect athletes’ procedural rights. In addition, the CAS typically does not investigate procedural shortcomings of first instance tribunals (since it has a *de novo* right of review, the CAS takes the stance that it can remedy any procedural failures at first instance on appeal).^148^ Unfortunately, this fails to remedy the fundamental issue of the failure to protect procedural rights in first instance disputes. The CAS has in these cases, therefore, missed an opportunity to set a precedent of what constitutes a violation of procedural fairness at first instance (and consequently what tribunals should be doing to protect such rights in the future). The amendments to the Code and the introduction of the ISRM have enshrined minimum procedural safeguards – however, in the interest of providing autonomy to NADOs and domestic panels, WADA has not provided a roadmap or “best practice” guidelines for doping tribunals in the same way that they have for testing authorities. Given the importance of protecting the procedural rights of athletes, coupled with the empirical evidence of systemic procedural failures at first instance, a mechanism for further accountability and institutional reform is necessary. WADA, with the input of NADOs, could prepare an international standard of doping tribunals that sets out “best practice” procedures and processes for first instance doping panels (whether conducted by federations or domestic bodies). This document would be more detailed than the broad requirements of the Code and the ISRM. It could, for example, include details of best practices in case management, milestone planning, and institutionalized support for athletes to remove access to justice barriers (including pro bono lists, or legal aid). While there would be challenges with revoking accreditation in the same way as testing labs (removing local first instance tribunals may actually increase access to justice barriers further), WADA could monitor and identify first instance tribunals that are consistently falling below these best-practice standards, and work with them to improve their procedures and protect procedural safeguards. The establishment of these “best practices” would show that WADA takes the rights included in the Athlete Anti-Doping Act, 2021 seriously, and acknowledge that there needs to be further investment in first instance procedures to ensure procedural consistency and the protection of athletes’ rights.

## Conclusion

Empirical research on anti-doping disputes can inform a productive debate about systemic procedural issues, and potential areas of reform. At the heart of WADA’s challenge in pursuing a harmonized system of anti-doping is the challenge of striking a balance between local legal culture and the need for uniformity to promote consistency and fairness. The empirical analysis conducted in this study highlights that while the Code purports to promote harmonization, there remain stark differences in terms of the implementation of anti-doping procedures at first instance. Most athletes who have been accused of an ADRV only have their allegations heard by domestic first instance tribunals, and it is critical that we understand the extent to which these domestic tribunals are complying with principles of procedural fairness prescribed by the applicable law. While scholars have previously argued the difficulties of achieving compliance with the procedural requirements of the Code,^149^ if institutions are serious about the quest for harmonization, future policy agendas need to be informed by empirical evidence,^150^ and strong monitoring of compliance needs to be backed by scientific integrity and data.^151^

This study highlights that there is clear evidence of delay in anti-doping procedures, particularly in India. This delay is apparent when the time taken to resolve ADRVs is compared to the prescribed time limits under the NADA Rules 2010, and when compared to the equivalent time taken to resolve similar anti-doping disputes in New Zealand and Canada. In addition, far fewer athletes are represented by legal counsel in India than in New Zealand and Canada. From the perspective of athletes, this is a troubling statistic, especially given athletes are more likely to receive a favorable outcome if they are represented by a lawyer than if they are self-represented. Within the anti-doping framework, WADA and NADOs are the only institutional “repeat players”, whereas athletes are “one shotters”, and as argued by Galanter, it is axiomatic that the “repeat players” with more resources and experience are far more likely to succeed on average. Accordingly, it is logical that the anti-doping system should seek ways to provide the “one shotters” – the athletes – institutional support, through education and stronger procedural safeguards.

To promote a level playing field, anti-doping rules and regulations are intentionally strict on athletes. Similarly, it is critical that procedural safeguards are strictly enforced against institutions. This is especially true given the imbalance of resources between anti-doping institutions and most athletes. Protecting the sanctity of procedural rights not only safeguards athletes but also upholds the legitimacy of the anti-doping framework.

## Footnotes

1 Soek and Siekmann (2012).

2 Star and Kelly (2021).

3 Kambhampati and Star (2021).

4 Star and Kelly (2021); Dasgupta (2019); Dimeo and Møller (2018); Efverström and Bäckström (2017).

5 Economides et al. (2013); Colvin (2011); Lederman and Hrung (2006).

6 Pielke Jr. and Boye (2019).

7 Star and Kelly (2021).

8 Dimeo and Møller (2018).

9 Star (2022); Mohan (2020); Sharma (2020).

10 Star (2022); NADA (2022a).

11 Star and Kelly (2021); Cernic (2014); Weston (2009).

12 Star and Kelly (2021); Dasgupta (2019).

13 See, for example, Lindholm (2019).

14 Star and Kelly (2021).

15 Read et al. (2019).

16 Galanter (1974). See also, He and Su (2013).

17 Star and Kelly (2021); David (2017).

18 Economides et al. (2013), p. 34; Heise (2000); Hodrick and Prescott (1997).

19 Viret (2020a, b); Pielke Jr. and Boye (2019).

20 See, WADA, “Code Signatories”, http://www.wada-ama.org/en/code-signatories. Note that under the United Nations Convention Against Doping in Sports (2005), the Code was adopted by the United Nations Educational Scientific and Cultural Organization (UNESCO) which was opened for signature on 19 October 2005 and became effective on 1 February 2007, http://www.unesco.org/new/en/social-and-human-sciences/themes/anti-doping/international-convention-against-doping-in-sport/.

21 It should also be noted that under the Olympic Charter, international sporting federations are specifically bound by the WADA Code pursuant to Rules 40, 43 and 45.3, and By-law to Rule 44.6.

22 NADA (2022a).

23 NADA (2022b).

24 See New Zealand Gazette, https://gazette.govt.nz/home/NoticeSearch?act=Sports+Anti-Doping+Rules. Accessed 6 December 2021.

25 Sports Anti-Doping Act 2006, s 38 (NZ).

26 Read et al. (2019).

27 Cane and Kritzer (2010); Cahoy (2010); Eisenberg (2004); Heise (1999); Schuck (1989).

28 Salehijam (2018); Rachlinski (2016); Bel (2016).

29 Galligan (2010).

30 Lindholm (2019).

31 Tahura and Kelly (2015); Cahoy (2010); George (2006).

32 Viret (2020b); Pielke Jr. (2018); de Hon et al. (2015).

33 Engelberg et al. (2012); Stamm et al. (2008); Bahr and Tjørnhom (1998); Scarpino et al. (1990).

34 Pielke Jr. and Boye (2019).

35 For a conceptual discussion of procedural fairness in anti-doping disputes, see Star and Kelly (2021).

36 Star and Kelly (2021).

37 NADA (2022a).

38 Star (2022); Star and Kelly (2021).

39 Heise (2000), p. 817.

40 Greiner and Pattanayak (2011); Sandefur (2010); Lederman and Hrung (2006).

41 Franck (2007): This study focuses on: “(1) who is involved in arbitration and what is arbitrated, (2) increases in awards, (3) win/loss rates, (4) amounts claimed and awarded, (5) arbitration costs, (6) use of other dispute resolution processes, and (7) nationality and gender of arbitrators”.

42 Colvin (2011).

43 The authors note that although some empirical research with respect to criminal procedure exists (see, e.g., Feld 1989), there is significantly more research in the context of civil litigation and administrative tribunals, applying quantitative research methodologies to analyse issues such as timeliness and access to justice.

44 Goerdt (1991); Luskin (1978); Chruch et al. (1978).

45 Economides et al. (2013); Cranston (1985); Grossman et al. (1981).

46 Economides et al. (2013).

47 Ibid; Heise (2000); Weatherburn and Baker (2000); Church (1982).

48 Economides et al. (2013); Sutton and Barwick (2000); Garner (1987); Selvin and Ebener (1984).

49 Righarts and Henaghan (2011); Marfording (2010).

50 Economides et al. (2013).

51 Ibid.

52 Dakolias (2014); Marfording (2010).

53 Sutton and Barwick (2000); Garner (1987); Selvin and Ebener (1984).

54 Economides et al. (2013).

55 Sandefur (2012), p. 40.

56 Lederman and Hrung (2006), p. 1239.

57 Ibid.

58 Sandefur (2010), p. 62.

59 Ibid, p. 67.

60 Ibid, p. 69

61 Colvin (2011).

62 Cf. Dakolias (2014).

63 NADA (2022a). It should be noted that the list published by NADA does not include cases where an athlete has tested positive for a banned substance and ADDP has cleared them of any violations of the NADA rules (such cases are not published by NADA).

64 Duval (2019); Hayes (2007).

65 Kambhampati and Star (2021).

66 In any event, there are a limited number of awards which have been handed down by first instance tribunals which apply the 2021 Code and ISRM, since it came into force on 1 January 2021. As such, limited data exists with which to compare and contrast whether the amended procedural requirements have had any impact in practice.

67 Pound and Clarke (2011), p. 152.

68 Kambhampati and Star (2021).

69 See Article 8 of the 2021 Code, and Article 4.2 and Article 8 of the ISRM.

70 See, Dispute Resolution Secretariat (Tribunal), http://www.crdsc-sdrcc.ca/eng/dispute-resource-databases-jurisprudence. Accessed on 6 December 2021.

71 See, Sports Tribunal of New Zealand, http://www.sportstribunal.org.nz/decisions/all-decisions/search. Accessed on 6 December 2021.

72 Star and Kelly (2021).

73 The only exception being cricket in India, or in some instances international federations may hear disputes involving international athletes. See Dasgupta (2021).

74 See, e.g., James Armstrong v. World Curling Federation (WCF) (CAS 2012/A/2756), award of 21 September 2012; World Anti-Doping Agency (WADA) v. International Waterski and Wakeboard Federation (IWWF) & Aaron Rathy (CAS 2012/A/2701), award of 21 November 2012; World Anti-Doping Agency (WADA) v. Angela Covert & Fédération Equestre Internationale (FEI) (CAS 2012/A/2960), award of 31 January 2014; Union Cycliste Internationale (UCI) v. Jack Burke & Canadian Cycling Association (CCA) (CAS 2013/A/3370), award of 17 July 2014; William Brothers v. Fédération Internationale de Natation (FINA) (CAS 2016/A/4631), award of 21 March 2017

75 This is especially true in developing countries such as India where only 14 athletes from more than 1000 ADRVs have appealed to the CAS (Star and Kelly 2021).

76 Note that four awards contained no information on the date of sample collection and were considered incomplete data sets for the purpose of this analysis.

77 The NADA Rules were revised in 2015 to reflect changes in the Code, and these strict time limits were relaxed, time limits were again amended as a result of the 2021 amendments to the Code and the ISRM (Article 4 and Article 8).

78 National Anti-Doping Rules, 2010 (India), Rule 8.3.8.1.

79 National Anti-Doping Rules, 2010 (India), Rule 8.3.8.2.

80 National Anti-Doping Rules, 2010 (India), Rule 8.3.8.3.

81 National Anti-Doping Rules, 2010 (India), Rule 8.3.7.

82 The researchers note that the 2010 NADA Rules are applicable to 566 cases in the sample and of these 10 cases had incomplete data and compliance could not be calculated for these cases (see Table 4). Therefore, compliance can be calculated with respect to 556 cases.

83 National Anti-Doping Rules, 2015 (India), Rule 8.4.

84 National Anti-Doping Rules, 2015 (India), Rule 8.3.9.

85 Star and Kelly (2021); Duval (2019).

86 See for example, (i) Bhupinder Singh v. NADA, Case 7/ADAP/2012, on 9 December 2012 (suspension lifted after 1 year and 4 month ban had already been served by the athlete); (ii) *Harpreet Singh v. NADA*, Case 7/ADAP/2014, on 6 August 2014 (the 6 month suspension was set aside on appeal after the athlete had served 1 month suspension); (iii) Monisha S. v. NADA, Case 27/ADDP/01/2014, on 16 February 2015 (sanction overturned on appeal at ADAP on 28 January 2016 after an 11.5 month suspension), cited in Star and Kelly (2021).

87 Christian Craig v. FIM, Decision of the FIM International Disciplinary Court, 29 July 2019, para 41.

88 ISRM (2021), Article 4.2.

89 NADA vs. Anil Kumar (Case No. 43.ADDP.01.2012).

90 Economides et al. (2013); Church (1982).

91 Sports Tribunal of New Zealand, “Rules of the Sports Tribunal of New Zealand 2012” (6 March 2012), r 30.

92 David (2016), p. 143.

93 See ISRM, Article 8, and the Code 2021, Article 8.

94 Star and Kelly (2021).

95 David (2016).

96 Gray (2019).

97 Rehn et al. (2011); Moog (1992).

98 Galanter and Krishnan (2004), p. 789.

99 Ibid.

100 David (2016).

101 Ibid, p. 141.

102 See e.g., Rules of the Sports Tribunal of New Zealand, 2012, Article 11(b) and 37 (which notes that pre-conferences are typically conducted via teleconference and that parties may consent to the pre-conference becoming the hearing).

103 Canadian Anti-Doping Program (2009), Rule 7.94; Canadian Anti-Doping Program (2015), Article 8.2.4(c); Canadian Anti-Doping Program (2021), Article 8.2.4.3.

104 Canadian Anti-Doping Program (2009), Rule 7.95(b); Canadian Anti-Doping Program (2015), Article 8.2.4(e); Canadian Anti-Doping Program (2021), Article 8.2.4.5.

105 NADA Rules, 2010 (India), Article 8.4.8 and Article 13.7.7; NADA Rules, 2015 (India), Article 8.3.14 and Article 13.2.2.2.8.

106 LawInSport (2020).

107 Levin (1975), p. 83.

108 WADA (2020); The Hindu 4 April 2017.

109 Lederman and Hrung (2006), p. 1270.

110 Sports Resolutions UK, http://www.sportresolutions.co.uk/services/pro-bono-legal-advice; Sports Tribunal of New Zealand, http://www.sportstribunal.org.nz/rules-and-procedures/legal/.

111 NADA Rules, 2010 (India), Article 8.4.6; NADA Rules, 2015 (India), Article 8.4.5.

112 Star and Kelly (2021), p. 107, citing Sports Resolutions UK, http://www.sportresolutions.co.uk/services/pro-bono-legal-advice; Sports Tribunal of New Zealand, http://www.sportstribunal.org.nz/rules-and-procedures/legal/.

113 Sandefur (2012).

114 For tax disputes, see: Lederman and Hrung (2006). For employment disputes, see: Colvin (2011).

115 Sandefur (2012); Lens (2007); Engler (1999); Conley and O’Barr (1990); O’Barr and Conley (1988).

116 Sandefur (2012), citing Kritzer (1998); O’Barr and Conley (1988); Hagen (1983).

117 Kritzer (1998), p. 83. In Kritzer’s (1998) study, there was a mixed outcome (partial win) in 27% of the counsel’s cases and 17% of unrepresented cases.

118 Colvin (2011).

119 Sandefur (2012), p. 44.

120 Ibid, p. 45.

121 World Anti-Doping Code: International Standard on Education, 2021, Article 5.2.

122 Galanter (1974); He and Su (2013).

123 Ibid.

124 Lederman and Hrung (2006), p. 1260.

125 Krishnan et al. (2014); Law Commission of India (1988).

126 Star and Kelly (2021).

127 Data provided to author by Head of Case Management at Sport Resolutions UK on 31 July 2020.

128 Colvin (2011), p. 16.

129 Ibid, p. 17.

130 David (2016), p. 144.

131 Article 17, Athlete’s Anti-Doping Rights Act, 2019. https://www.wada-ama.org/sites/default/files/resources/files/athlete_act_en.pdf.

132 Gray (2019); Houlihan (2013).

133 Cernic (2014).

134 Lindholm (2019), p. 16.

135 Bersagel (2012).

136 NADOs are not required by WADA to publish full-text awards of first instance disputes under the Code. However, under Article 14 of the Code, NADOs are only required to publicly disclose certain details where an athlete has committed an ADRV, which is important for public transparency and the enforcement of sanctions. Details typically include *inter alia*, the athlete’s name, country, sport, competition level, type and nature of ADRV, and length of sanction. It is therefore a policy decision of each jurisdiction to determine whether they will publish full-text awards for each ADRV (in addition to the minimum public disclosure requirements).

137 WADA (2021a).

138 Müller (2017), p. 186.

139 WADA (2016).

140 Rooze (2010).

141 Ibid.

142 For instance, as discussed above data from Sport Resolutions UK suggests that since 2017, more than 96% of athletes have been represented, with more than 60% of all athletes having been represented by counsel on a pro bono basis.

143 Article 17, Athlete’s Anti-Doping Rights Act, 2019. https://www.wada-ama.org/sites/default/files/resources/files/athlete_act_en.pdf.

144 See e.g., the USOPC Athlete Ombuds in the USA, details available at https://www.usada.org/athletes/usopc-athlete-ombuds/.

145 Sandefur (2012), p. 46.

146 See, Código Brasileiro de Justiça Desportiva, Article 31 (Brazilian Code of Sports Justice). These attorneys represent athletes on a pro bono basis.

147 WADA (2021b).

148 See, Star and Kelly (2021).

149 Star and Kelly (2021).

150 Viret (2020a).

151 Pielke Jr. and Boye (2019).
